# Investigation on the differences of hemodynamics in normal common carotid, subclavian, and common femoral arteries using the vector flow technique

**DOI:** 10.3389/fcvm.2022.956023

**Published:** 2022-11-17

**Authors:** Di Song, Mengmeng Liu, Yinghui Dong, Shaofu Hong, Ming Chen, Yigang Du, Shuangshuang Li, Jinfeng Xu, Wenjing Gao, Fajin Dong

**Affiliations:** ^1^Department of Ultrasound, Shenzhen People’s Hospital (The Second Clinical Medical College, Jinan University, The First Affiliated Hospital, Southern University of Science and Technology), Shenzhen, Guangdong, China; ^2^Shenzhen Mindray Bio-Medical Electronics Co., Ltd., Shenzhen, Guangdong, China

**Keywords:** vector flow imaging (V Flow), ultrasound, common carotid arteries (CCA), subclavian arteries (SCA), common femoral arteries (CFA), wall shear stress (WSS), turbulence index (Tur)

## Abstract

**Objectives:**

To investigate the feasibility of the vector flow imaging (V Flow) technique to measure peripheral arterial hemodynamic parameters, including wall shear stress (WSS) and turbulence index (Tur) in healthy adults, and compare the results in different arteries.

**Materials and methods:**

Fifty-two healthy adult volunteers were recruited in this study. The maximum and mean values of WSS, and the Tur values at early-systole, mid-systole, late-systole, and early diastole for total 156 normal peripheral arteries [common carotid arteries (CCA), subclavian arteries (SCA), and common femoral arteries (CFA)] were assessed using the V Flow technique.

**Results:**

The mean WSS values for CCA, SCA, and CFA were (1.66 ± 0.68) Pa, (0.62 ± 0.30) Pa, and (0.56 ± 0.27) Pa, respectively. The mean Tur values for CCA, SCA, and CFA were (0.46 ± 1.09%), (20.7 ± 9.06%), and (24.63 ± 17.66%), respectively. The CCA and SCA, as well as the CCA and CFA, showed statistically significant differences in the mean WSS and the mean Tur (*P* < 0.01). The mean Tur values had a negative correlation with the mean WSS; the correlation coefficient between log(Tur) and WSS is −0.69 (*P* < 0.05).

**Conclusion:**

V Flow technique is a simple, practical, and feasible quantitative imaging approach for assessing WSS and Tur in peripheral arteries. It has the potential to be a useful tool for evaluating atherosclerotic plaques in peripheral arteries. The results provide a new quantitative foundation for future investigations into diverse arterial hemodynamic parameters.

## Introduction

Peripheral arterial disease (PAD) is atherosclerosis of the peripheral arteries that is linked to a higher risk of stroke, arterial dissection, and myocardial infarction, making it a severe medical challenge worldwide (1–3). According to global data, the number of individuals suffering with PAD has increased to 236 million, the proportion of amputations has risen to 18%, and the 5-year mortality rate for severe chronic PAD has risen to over 70% (4, 5). Many patients are unaware of their condition and unable to obtain timely and effective treatment, especially in low-income countries, where the prevalence of peripheral artery disease is increasing as the population ages (6). Early detection of PAD hemodynamics is therefore crucial for disease prevention. Vascular elasticity and arterial stiffness, which can be measured using strain imaging, shear wave elastography (SWE) and pulse wave velocity (PWV), are considered to be strongly related to atherosclerosis, but they mainly represent the current status of blood vessels and plaques, and sometimes are limited by the measurement accuracy on the vessel wall (7, 8).

Wall shear stress (WSS) is considered the frictional force acting on the surface of endothelial cells during blood flow, which is clinically significant in atherosclerosis research (9, 10); high WSS is associated with high-risk plaque features, and intraplaque hemorrhage in asymptomatic carotid plaques (11, 12); moderate WSS (physiologically high WSS) protects against atherosclerosis by suppressing prothrombotic tissue and anti-inflammatory activity of endothelial cells (13–15); low WSS is prevalent in the prone area of atherosclerosis, stimulating the atherogenic phenotype (16), and thus promotes the formation and progression of atherosclerotic plaques, whereas a specific range of WSS inhibits plaque formation (13, 17). Furthermore, studies have revealed that there are different normal WSS thresholds in peripheral arteries such as carotid and femoral arteries, but there are no consistent criteria to quantify peripheral arteries in these studies, and the methods to measure WSS vary, mainly magnetic resonance imaging (MRI), computed tomography (CT), and ultrasound (18–22). MRI is costly, time-consuming, and has limited spatial resolution, making accurate measurements of velocities adjacent to the vessel wall challenging. CT has a high radiation exposure. Neither of them can be used on a large scale by the public.

The high-frame rate dynamic displayed vector flow imaging (V Flow) technique is a new ultrasonic vector flow approach that is easy and feasible for measuring WSS (14, 23, 24). V Flow technique can obtain the actual vector velocities reconstructed by measuring the velocity components at different ultrasound transmission angles, enabling more precise blood velocity and blood flow measurements (25, 26). Traditional pulsed-wave Doppler (PW-Doppler) quantitative blood flow velocity measurements can only detect laminar flow and need angle adjustment. It is not able to measure non-laminar flow velocity values due to the impossible angle correction as it cannot measure the correct blood velocity direction for intricate blood flows such as turbulence and vortex. Meanwhile, V Flow can use Tur values to minimize heterogeneity in the evaluation by quantifying the degree of dispersion of complicated blood flow in a certain spatial range, such as vortex or turbulence (23). V Flow provides a more accurate and realistic evaluation of the magnitude, direction, and degree of dispersion of complex blood flow than conventional methods of assessing peripheral artery hemodynamics utilizing color Doppler and PW-Doppler to calculate blood flow velocity and volume flow (25).

Efficient, accurate, and convenient assessment of peripheral arterial hemodynamics and early detection of PAD is one of the important indications for the prevention of atherosclerotic plaques. The purpose of this study was to use the V Flow technique to evaluate common carotid arteries (CCA), subclavian arteries (SCA), and common femoral arteries (CFA) hemodynamic parameters, including wall shear stress (WSS) and turbulence index (Tur) in healthy adults, and compare the results in different arteries.

## Materials and methods

### Ethics

The Ethics Committee of Shenzhen People’s Hospital approved this prospective study (approval no: LL-KY-2021685), and all volunteers provided informed consent. 52 healthy adult participants were recruited from July 2021 to January 2022.

### Subjects

Age > 18 years, normal blood pressure, blood sugar, and cholesterol levels in the month before the examination were the criteria for inclusion. Exclusion criteria included: (1) severe systemic diseases; (2) previous diabetes, hypertension, hyperlipidemia, and other cardiovascular diseases; (3) the existence of arterial plaque or intima-media thickness (IMT) > 1 mm in the CCA, SCA, or CFA; (4) BMI > 24; and (5) poor quality ultrasound images. Volunteers’ gender, age, and serological indicators were obtained.

### Ultrasound equipment

All ultrasound examinations were done using a Mindray Resona 7 diagnostic ultrasonography machine (Shenzhen, China) with a line array probe (L11–3U) and an installed V Flow fixation procedure. WSS and Tur measurements are the sub-functions of V Flow and implemented based on measured vector velocities (14, 23).

### Ultrasound V Flow analysis

**FIGURE 1:**
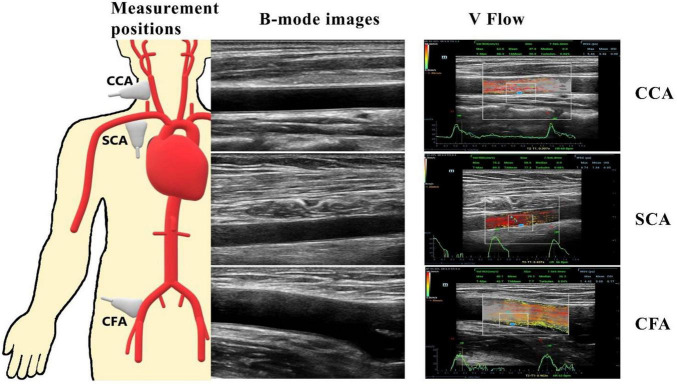
CCA, common carotid artery at 20 to 30 mm of the carotid bifurcation; SCA, subclavian artery at 25 to 35 cm from the start of the SCA; CFA, common femoral artery at 15 to 25 mm of the common femoral artery bifurcation. The pictures on the right represent two-dimensional ultrasound images of the three peripheral arteries and a vector flow imaging technique (V Flow) to measure peripheral arterial hemodynamic parameters, including wall shear stress (WSS) at the place where the blue dot locates and the turbulence index (Turbulen) in the small ROI.

Volunteers were asked to lie down with their heads inclined back to expose the necks, thighs abducted and externally rotated, and knees slightly flexed. The IMT of the CCA, SCA, and CFA were measured in longitudinal sections at 20 to 30 mm from the carotid artery bifurcation, 25 to 35 cm from the beginning of the SCA, and 15 to 25 mm from the bifurcation of the CFA, respectively (Figure 1). The PSV was measured for the three arteries using the conventional PW-Doppler (23), at a pulse repetition frequency (PRF) around 4 to 5 kHz depending on the imaging depth and maximum velocity, and at an incidence angle from 45 to 60° depending on the actual slope of the blood vessel. Note that the PSV was not recorded by V Flow since it was implemented with the different technique compared to the conventional PW. A comparison study demonstrated that PSV using PW was overestimated compared to the measurements of V Flow and MRI (25). However, the PW is still used in the current diagnostic guidelines of the related vascular disease due to its highly reproducible measurements (27).

V Flow employs multi-angle Doppler transmission and reception (23, 26), and achieves a high frame rate flow imaging (FR: 374∼1,240 Hz) for dynamic display using interleaved transmissions with plane wave and focus wave, which also ensures a high spatial resolution B-mode image simultaneously (23). The vector velocity is obtained by combining all velocity components from different steering angles (26). The frame rate is linked to the PRF, which can be adjusted to meet the user’s needs, with the goal of achieving high velocity blood flow without aliasing with a set depth of 3–4 cm. After clearly displaying the artery wall structure of CCA, SCA, and CFA in V Flow mode, dynamic V Flow data were acquired by clicking the “Update” button. The region of interest was analyzed for 1.5 s and encompasses at least one cardiac cycle. Image sequences were generated automatically from the data. The color vector representing the velocity and direction of each point in the artery reflects the blood flow signal. Blood flow velocities are represented by different colors. The blood flow velocity is likewise proportional to the length of the arrow; the longer the arrow, the higher the blood flow velocity. A full cardiac cycle was set, the maximum and average values of posterior wall WSS were recorded at CCA, SCA, and CFA, respectively, while the Tur value was measured at early-systole, mid-systole, late-systole, and early diastole, and the ROI was chosen to be around 3 × 2 cm in size (Figure 1). Tur values range from 0 to 1, with values closer to 0 representing laminar flow and values closer to 1 reflecting more turbulent blood flow (23).

### Statistical analysis

The mean standard deviation (SD) was used to express continuous variables. Differences between peripheral arteries were tested with the ANOVA or Kruskal-Wallis test (28). Comparisons between two groups were evaluated using independent-sample *t* tests or *t*‘ tests. The relationship between WSS values among different peripheral arteries was illustrated by Spearman’s correlation coefficient. The statistical analysis was performed with R version 3.6.2^1^ and the MATLAB program (The MathWorks, Natick, MA, USA).

## Results

### Population characteristics

A total of 156 ultrasound images of normal peripheral arterial structures were acquired from 52 healthy volunteers, 22 males and 30 females, with a mean age of 42 ± 13 years old. The detailed clinical information for the enrolled population is shown in Table 1.

**TABLE 1:** Characteristics of the volunteers (*n* = 52).

Characteristics	Mean (SD) or proportion (%, n)
Male	42% (22)
Age, years	42 (13)
BMI, kg/m^2^	22.62 (3.39)
HDL-C, mmol/L	1.43 (0.31)
LDL-C, mmol/L	3.06 (0.90)
Triglycerides in mmol/L	1.07 (0.53)
Total cholesterol, mmol/L	4.8 (1.07)
Fasting plasma glucose, mmol/L	4.75 (0.45)
IMT, mm	< 1
CCA	0.63 (0.10)
SCA	0.67 (0.11)
CFA	0.70 (0.15)

HDL-C, high-density lipoprotein cholesterol; LDL-C, low-density lipoprotein cholesterol; SD, standard deviation; IMT, intima-media thickness; CCA, common carotid artery; SCA, subclavian artery; CFA, common femoral artery.

### The PSV of common carotid arteries, subclavian arteries, and common femoral arteries by pulsed-wave-Doppler

**FIGURE 2:**
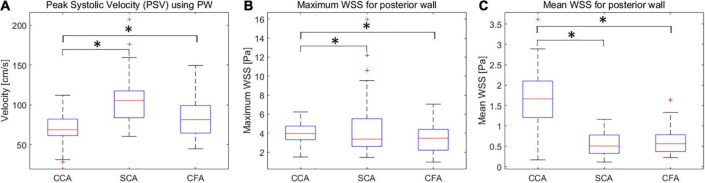
Box plots of PSV **(A)**, wall shear stress (WSS) maxima **(B)** and means **(C)** for CCA, SCA, and CFA in healthy adult volunteers (vertical axis) corresponding to different peripheral artery locations (horizontal axis). CCA, common carotid artery; SCA, subclavian artery; CFA, common femoral artery. *P < 0.05.

The mean PSV values for CCA, SCA, and CFA were (70.9 ± 17.3) cm/s, (105.6 ± 30.5) cm/s, and (84.2 ± 24.1) cm/s, respectively. Among CCA, SCA, and CFA, the PSV was statistically significant (P < 0.01) (Figure 2A).

### Normal wall shear stress values of common carotid arteries, subclavian arteries, and common femoral arteries

The maximum WSS values for CCA, SCA, and CFA were (4.03 ± 1.02) Pa, (3.51 ± 1.44) Pa, and (4.40 ± 2.85) Pa, respectively; the mean WSS values were (1.66 ± 0.68) Pa, (0.62 ± 0.30) Pa, and (0.56 ± 0.27) Pa. Among CCA, SCA, and CFA, the mean WSS value was statistically significant (*P* < 0.01 for CCA vs. SCA, and CCA vs. CFA). However, the mean WSS values of SCA and CFA were not significantly different (*P* = 0.45) (Figures 2B,C). It was different from the comparison for the mean WSS since the mean PSV in SCA is higher than those in CFA and CCA.

### The performance of Tur in common carotid arteries, subclavian arteries, and common femoral arteries

**FIGURE 3:**
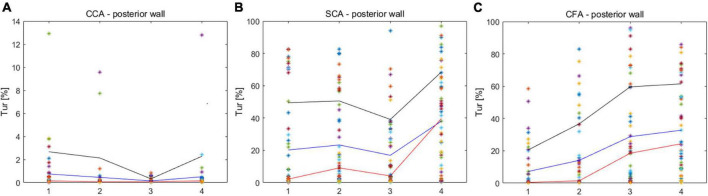
Turbulence index (Tur) values corresponding to different locations in early-systole, mid-systole, late-systole, and early diastole. **(A)** CCA corresponds to the Tur value of four stages; **(B)** SCA corresponds to the Tur value of four stages; **(C)** CFA corresponds to the Tur value of four stages. CCA, common carotid artery; SCA, subclavian artery; CFA, common femoral artery.

The Tur values with the longest duration in early-systole, mid-systole, late-systole, and early diastole were recorded after a complete cardiac cycle was set in the V Flow data obtained in CCA, SCA and CFA (Figure 3).

**FIGURE 4:**
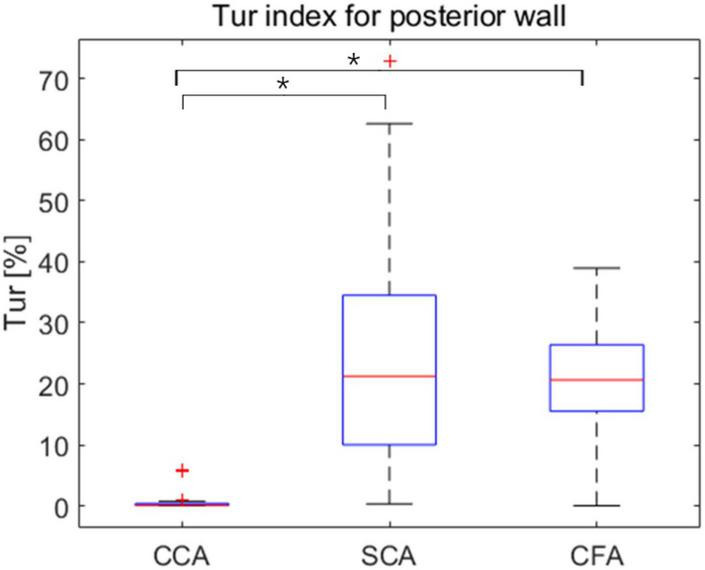
Box plots exhibit mean turbulence index (Tur) values at three locations during early-systole, mid-systole, late-systole, and early diastole, with mean Tur values in healthy adult volunteers (vertical axis) corresponding to three sites (horizontal axis). CCA, common carotid artery; SCA, subclavian artery; CFA, common femoral artery. *P < 0.05.

The mean Tur values for CCA, SCA, and CFA were (0.46 ± 1.09%, (20.7 ± 9.06%), and (24.63 ± 17.66%), respectively, and the difference in mean Tur values for the three arteries was statistically significant (*P* < 0.01 for CCA vs. SCA, and CCA vs. CFA) but the mean Tur values of SCA and CFA were not significantly different (*P* = 0.52) (Figure 4).

### Correlation of the mean wall shear stress values and the mean Tur values in common carotid arteries, subclavian arteries, and common femoral arteries

**FIGURE 5:**
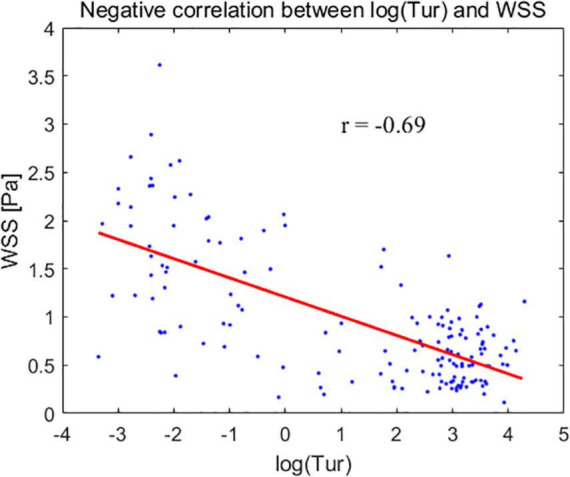
The modified mean values of turbulence index (Tur) – log(Tur) (horizontal axis) for the common carotid artery, subclavian artery, and common femoral artery correspond to the mean wall shear stress (WSS) (vertical axis).

The mean Tur values had a negative correlation with the mean WSS. The correlation coefficient is −0.69 (*P* < 0.05), when the modified mean values, log(Tur) were used to compared to the mean WSS measurements (Figure 5).

## Discussion

The innovative V Flow technique was used to assess the hemodynamic characteristics of 156 normal peripheral arteries in CCA, SCA, and CFA, including WSS maximum and mean values during one cardiac cycle, and Tur values during early systole, mid-systole, late systole, and early diastole, respectively. To the best of our knowledge, this is the first study of both WSS and Tur in peripheral arteries (i.e., CCA, SCA, and CFA) that may be utilized as a reference standard for future clinical research of peripheral arteries.

We found that the mean WSS of the CCA was higher than that of the SCA and CFA, showing that the mean WSS of different locations of the peripheral arteries has a significant difference. The difference in WSS reflected the difference in atherosclerosis risk, with CFA having much lower mean WSS, indicating that the degree of atherosclerosis in CFA was higher than in CCA (29, 30). This is consistent with the reports of Stroev et al. and Wu et al., who used MRI to measure the results (20, 21). Nevertheless, the CFA supplies the lower limbs mostly, whereas the CCA primarily supplies the brain. Therefore, the CCA is more prone to resulting in adverse consequences like stroke when plaque stenosis or shedding occurs. WSS was shown to be linked to vessel wall shape and flow state (13, 24, 30, 31). The CCA, SCA, and CFA were all flat arteries with consistent vessel wall morphology, implying that the discrepancies in WSS in this research were due to the different hemodynamics of the peripheral arteries. It is thought to be linked to different resistance indices in various peripheral artery locations (32). The WSS of CCA measured in this study was higher than that obtained by Qiu et al. (24). The key distinction was thought to be the varied measurement sites. They were measured at 10 mm from the start of the CCA segment, or at their midpoint. In contrast, the measuring site in our study was 20 to 30 mm from the carotid bifurcation.

Common carotid arteries (CCA), subclavian arteries (SCA), and common femoral arteries (CFA) exhibited significant variations of Tur during early systole, mid-systole, late systole, and early diastole among healthy adult volunteers. All four periods of CCA were dominated by laminar flow. Despite the fact that SCA was laminar-dominated in systole, the degree of Tur increased in early diastole, demonstrating a rather unstable and complicated blood flow condition. Although the degree of disturbances in SCA was significantly higher than in CCA, the trend of disturbance changes in the four phases of CCA and SCA was similar, which may be associated with the fact that both CCA and SCA originate from the brachiocephalic artery. However, the angle formed between the SCA and the brachiocephalic artery was larger than that formed by the CCA, which might be the reason for the hemodynamic disturbance. CFA displays laminar flow during the early systolic period, and the Tur rises from late systole to early diastole, which is not the situation as with CCA. The creation of reverse blood flow creates an increase in the Tur because the blood flow velocity of CFA falls fast after peak systole. Overall, the degree of hemodynamic disturbance was significantly higher in SCA and CFA than in CCA in one cardiac cycle of healthy adult volunteers; the changes of hemodynamic disturbance were different in different phases of SCA and CFA; the changes of hemodynamic disturbance were similar in different phases of CCA and SCA, though the average disturbance was much lower in CCA than in SCA.

Plaque formation usually occurs in non-laminar regions (33–35). Tur values increase with the degree of blood flow disturbance and arterial stenosis (23, 36). Low WSS or high oscillatory WSS affect the morphological structure and function of endothelial cells, as well as promote the formation of atherosclerosis (16, 37, 38). This implies that the lower the WSS, the higher the Tur value and the greater the likelihood of atherosclerotic plaque formation. In our study, the mean Tur values of peripheral arteries were negatively correlated with the mean WSS, with a correlation coefficient of −0.69 (*P* < 0.05). The non-laminar flow with high Tur values, including vortex and turbulent flows also indicates a low momentum of fluid which is usually accompanied with low WSS (14, 16, 39). This could be the reason why the two parameters (WSS and Tur) are negatively correlated since both low WSS and non-laminar flow are related to atherosclerosis and plaque formation. A previous study (36) demonstrated that flows with Tur value < 1% can be considered laminar or nearly laminar flow. Another recent study (40) presented the Tur difference between common carotid artery and carotid bulb. In both two studies, it can be seen that quantification for the flow turbulence using Tur index to distinguish laminar and non-laminar flows is not a linear variation trend since for non-laminar flows Tur values range from 1 to 100%, which is much greater than the laminar flow range of 0 to 1%. Therefore, we used log(Tur) to study the linear correlation between Tur and WSS results.

Several limitations of the research can be noted. The current study focused on the advanced hemodynamics, and the related elastic parameters of peripheral arteries, e.g., vascular strain, SWE or PWV, were not considered. The study had a small sample size and targeted at healthy adults, although we evaluated more peripheral arteries than in earlier investigations. As V Flow is a two-dimensional imaging technique that cannot detect blood flow velocity outside the imaging plane, the accuracy of the corresponding WSS and Tur measurements will be affected. Meanwhile, only four time phases of Tur were chosen, which cannot accurately depict the trend of Tur, but this study has taken the first step toward quantifying the degree of dispersion of peripheral artery hemodynamics as an investigation of the novel approach of V Flow.

## Conclusion

The WSS and Tur of peripheral arteries differ greatly from one location to the next. Hence, using a single threshold to forecast the trend of lesions in all peripheral arteries is erroneous, and it should be assessed in light of current peripheral artery characteristics. The outcome of measuring and presenting the hemodynamic parameters of CCA, SCA, and CFA, as well as their associated thresholds, were presented in this study and may be used as a reference for future research on the prevention and progression of peripheral artery disease. Overall, V Flow is a simple, practical, and feasible quantitative imaging approach for assessing WSS and Tur in peripheral arteries, and it has the potential to be a useful tool for evaluating atherosclerotic plaques in peripheral arteries.

## Data availability statement

The raw data supporting the conclusions of this article will be made available by the authors, without undue reservation.

## Ethics statement

The studies involving human participants were reviewed and approved by the Ethics Committee of Shenzhen People’s Hospital (approval no: LL-KY-2021685). The participants provided their written informed consent to participate in this study.

## Author contributions

DS, ML, JX, and FD: study concept and design. DS, ML, YhD, SH, and WG: acquisition of data. DS, MC, and YgD: analysis and interpretation of data. DS and YgD: drafting of the manuscript. JX and FD: critical revision of the manuscript for important intellectual content. JX, FD, YgD, and SL: administrative, technical, or material support, and study supervision. All authors read and approved the manuscript and had access to the study data.

## Footnotes

1
http://www.r-project.org
